# Supplementary material for: Puzzles in modern biology. I. Male sterility, failure reveals design
Source: F1000Res. 2016 Sep 9;5:2288. [Version 1] doi: 10.12688/f1000research.9567.1 (PMC5147525; doi:10.12688/f1000research.9567.1)
Supplement: Peer review history

# Peer review: Referee response for version 1

**DOI:** 10.5256/f1000research.10304.r18319

**Published:** 2016-12-07

**Authors:** Harmit S. Malik

**Affiliations:** 1 Fred Hutchison Cancer Research Center, Seattle, WA, USA

This is a thoughtful and (characteristically) thought-provoking article on why sterility is a frequent observation in natural populations, despite all expectations to the contrary. The author cites a number of case studies to point out two basic underlying principles- the first being conflict and coevolution for instance between ‘male-harming’ mitochondria (my emphasis) and nuclear suppressors of male-harming behaviour. This arises from asymmetry of inheritance of nuclear and mitochondrial genomes. A related asymmetry of sex chromosomes gives rise to differential rates of evolution and therefore different opportunities for mismatches in gene expression, for instance seen in hybrids. The theme of gamete-killing meiotic drivers and their suppressors is not limited to sex chromosomes however, and a brief mention of fungal spore-killers and their suppressors would nicely round out the discussion. Finally such co-adapted systems rely on co-inheritance for maximum manifestation and as such, propagate best when recombination is not allowed to break up the co-adapted complex. Thus, many of these drive systems arise and are spurred by chromosome rearrangements that further imperil proper meiotic recombination and fertility.

 A second principle that might emerge is intrinsic, whereby for instance male sterility might, under the right circumstances, be favoured to encourage outcrossing.

 Ultimately, the author espouses the view that failure in a process where success is expected reveals the design principles. Much in the same way a genetic screen might reveal the underlying components (an excellent allegory to this is “The salvation of Doug” by Bill Sullivan.... http://sullivan.mcdb.ucsc.edu/salvation-doug.html). However, it is here that I feel the analogy falters a bit.

 Despite my discomfort at the use of the term “design”, I feel the author refers to two different things as design. The first is about the genetic principles by which a pathway works (“cutting a brake wire in a car”). The second is that of selfish genetic elements, that have no socially redeeming value to contribute to a biological process, but exist and thrive solely due to their ability to exploit weaknesses (like a petulant hacker overriding the car’s electronic controls at the most inopportune time). While the first definition is about natural selection refining a biological process, the second is about loopholes that exist and likely will always remain, which sponsor ‘selfish behaviour’ in genes even at the expense of important attributes of the biological system, such as fertility.

I have read this submission. I believe that I have an appropriate level of expertise to confirm that it is of an acceptable scientific standard.

# Peer review: Referee response for version 1

**DOI:** 10.5256/f1000research.10304.r16210

**Published:** 2016-09-13

**Authors:** Andy Gardner

**Affiliations:** 1 School of Biology, University of St Andrews, St Andrews, UK

This is a snappy and thought-provoking Opinion article, written in Steven Frank’s characteristically laconic style.

 The main message of the article is that, by investigating apparent failures in the design of organisms, we get a deeper understanding of organismal design. The particular focus here is on the suprisingly high levels of male sterility observed in many populations, and Frank takes us on a whirlwind tour of sperm dysfunction, pollen abortion and hybrid male sterility, highlighting commonalities and providing synthesis.

 Frank ends the article with a compelling metaphor: a car is a complicated object whose design may be difficult to fathom simply by inspection; but, by cutting a wire and noting that it causes the brakes to fail, we learn something about how it is put together. The design of the organism is also illuminated by its failure.

 I agree with all this, but would add two further thoughts.

 First, it could be that apparent failure owes not to actual maladaptation on the organism’s part but rather a misunderstanding on our part as to what constitutes good design. For example, sterile individuals abound in social insect colonies, and contemplation of this apparent puzzle led to a much deeper understanding of what it is organisms are designed to do (the theory of inclusive fitness).

 Second, it could be that failure reveals we have been looking for design in the wrong place. Several of the specific examples discussed in this article concern conflicts of interest within the genome, e.g. between mitochondrial vs nuclear genes. Failure might not reveal organism design, but design at the gene level.

 As is my policy, I waive anonymity

 Andy Gardner

I have read this submission. I believe that I have an appropriate level of expertise to confirm that it is of an acceptable scientific standard.
